# The BULT Method for Pediatric Minilaparoscopic Pyeloplasty in Infants: Technique and Results

**DOI:** 10.3389/fped.2016.00054

**Published:** 2016-05-25

**Authors:** Barbara Magda Ludwikowski, Michael Botländer, Ricardo González

**Affiliations:** ^1^Pediatric Surgery and Urology, Auf der Bult Kinder- und Jugendkrankenhaus, Hannover, Niedersachsen, Germany

**Keywords:** ureteropelvic obstruction, infants, pediatric, hydronephrosis, laparoscopy, pyelopalsty

## Abstract

We reviewed retrospectively the results of transperitoneal minilaparoscopic pyeloplasty in children younger than 2 years. The surgical technique utilized as well as the retrograde placement of the stent is described in detail. Twenty-four consecutive children with a mean age of 7.9 months (range 1–23), a mean weight of 7.4 kg (range 4–12), and a mean follow-up of 18 months (range 3–59) are included. Preoperative grade of dilatation was 3.8 (SFU scale) and postoperatively improved to 1.5. The AP diameter of the pelvis decreased from a mean of 28–9 mm. In 83% of cases, there was complete resolution of hydronephrosis (grades 0–2) and the rest showed improvement. There was one conversion to open surgery in a child with a horseshoe kidney. There was one omental *prolapse* though a port site in a child in whom an inappropriate drain was used. There were no stent-related complications and no reinterventions for persistent or recurrent obstruction. Given these outcomes, low complication rate and excellent cosmetic results, we recommend transperitoneal minilaparoscopy with a double J stent and a perirenal drain for infants requiring pyeloplasty.

## Introduction

Laparoscopic pyeloplasty (LP) is well established as the preferred method to treat ureteropelvic junction obstruction (UPJO) in adults and older children. Minilaparoscopy (MLP) refers to the use of small (i.e., 3 mm diameter, 20 cm in length) instruments suitable for work in small children. In some institutions, including ours, MLP is applied to children of all ages (1). Nevertheless, open surgery continues to be preferred for younger children in some centers (2, 3). In a recent survey, including children operated in the USA between 2004 and 2008, only 3.6% of children with UPJO were treated laparoscopically, underlining the technical difficulties of LP except in the hands of a few. Here, we report a standard method of MLP that we evolved over the last 15 years (4), which has been applied at our institution since 2010 and is particularly suited for small children. Since our method appears to differ substantially from widespread practices in several regards and our complication rate is low, we describe the technique in detail and the results obtained in 24 consecutive infants <2 years of age.

## Materials and Methods

Retrospective record review of all children undergoing pyeloplasty at our institution since 2010. Institutional ethical approval was obtained. Patient demographics, weight, form of presentation, and outcomes were recorded. Pre- and postoperative imaging studies were reviewed. Indications for MLP included persistent significant or worsening hydronephrosis detected pre- or neonatally, hydronephrosis, decreased differential function in renogram (40% or less), or repeatedly obstructed curves on the diuresis renogram. Grading of hydronephrosis was by the SFU classification (5, 6) and anterior posterior pelvic diameter measured on transverse posterior views on sonography.

### Method of Operation

**Figure 1:**
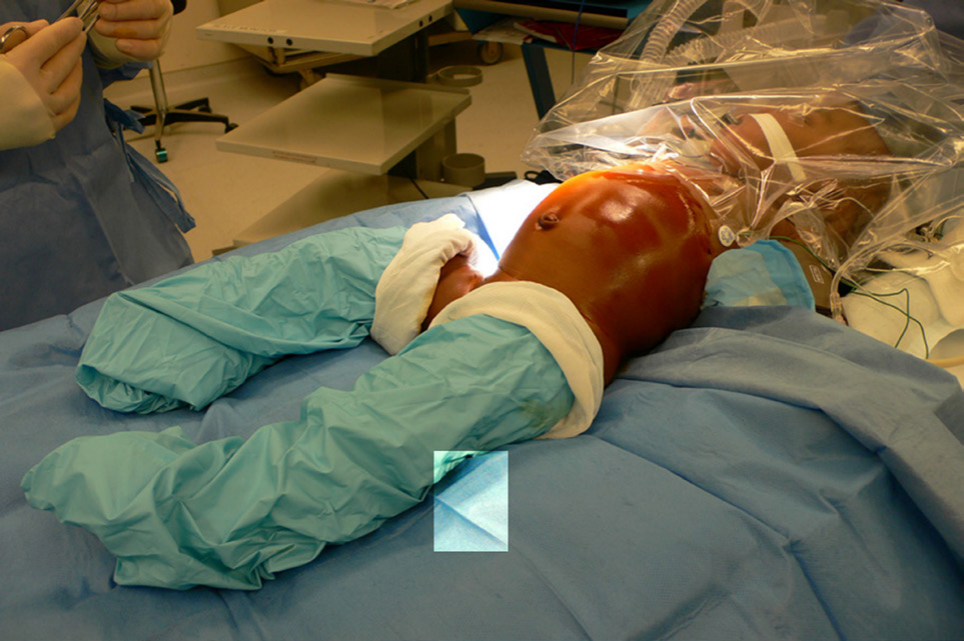
**Total body prep allows cystoscopic placement of double J stent and laparoscopic procedure without re-draping**.

A total body prep is performed and the cystoscopy and laparoscopic procedures are done without having to re-position or re-prep the patient (Figure 1). The procedure starts with a cystoscopy, a retrograde uretero-pyelogram and retrograde placement of a double J stent. For this purpose, a 3 or 4 Ch. open-ended ureteral catheter is inserted a few centimeters into the ureter of the affected side. Contrast medium is injected under fluoroscopy and the images saved. The catheter is then rinsed with normal saline and the guide wire provided in the kit inserted in the renal pelvis under fluoroscopic control. When the available guide wire cannot be passed to the pelvis, the use of a hydrophilic wire is usually successful. A double J stent (JJ) 3.7 Ch. 12 or 14 cm long is inserted in the renal pelvis. If the wire or stent cannot be advanced to the pelvis, they are left in the proximal ureter to be retrieved during the MLP procedure or to aid in the antegrade placement of a stent. They are secured to the bladder catheter.

**Figure 2:**
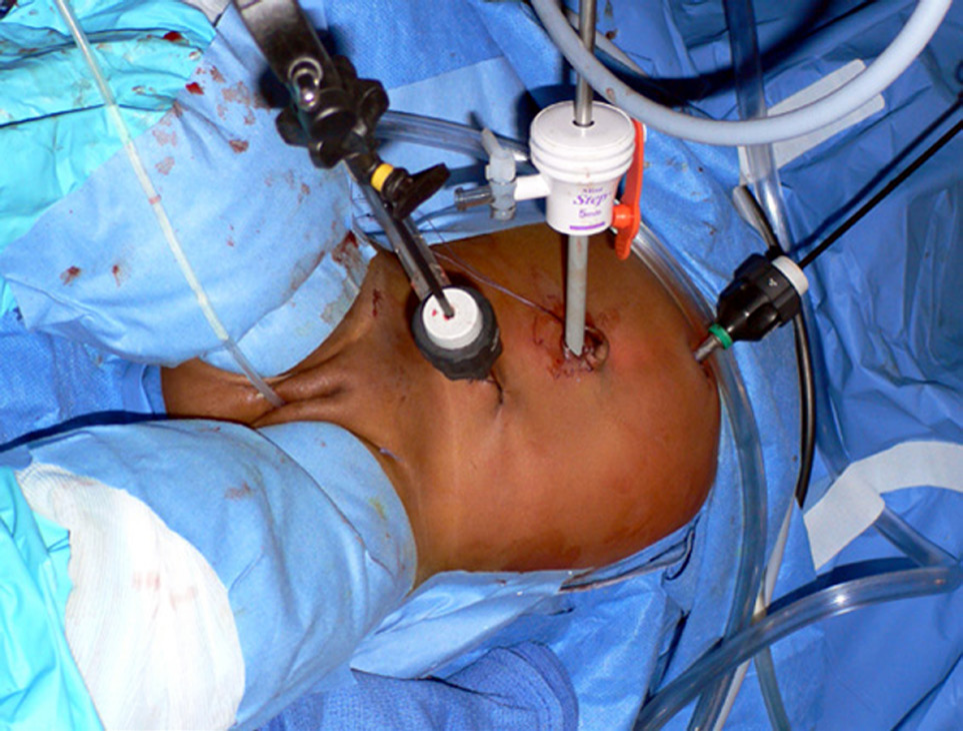
**Placement of the ports for left-sided pyeloplasty**.

The port placement is shown in Figure 2. A 5-mm disposable trocar^1^ for the lens is placed in the umbilicus using the Bailez technique (7). The choice of trocar is important to allow the introduction of the sutures into the abdomen. Two additional re-usable 3–5 mm^2^ ports are placed, one near the midline, half way between the umbilicus and the xiphoid on the side of the falciform ligament corresponding to the affected kidney and in the other in the corresponding lower quadrant. Twenty-centimeter-long, 3-mm-diameter instruments are used. Monopolar coagulation is used with discretion. The cutting function of electrosurgical unit is turned off.

After placement of the ports, the bladder catheter is clamped to allow distention of the renal pelvis. On the right-sided cases, the hepatic flexure of the colon is mobilized. The ureter is identified high in the retroperitoneum and followed toward the pelvis. The UPJ is dissected using coagulation only as strictly necessary. On the left side, whenever possible the approach to the renal pelvis is trans-mesocolonic (8).

**Figure 3:**
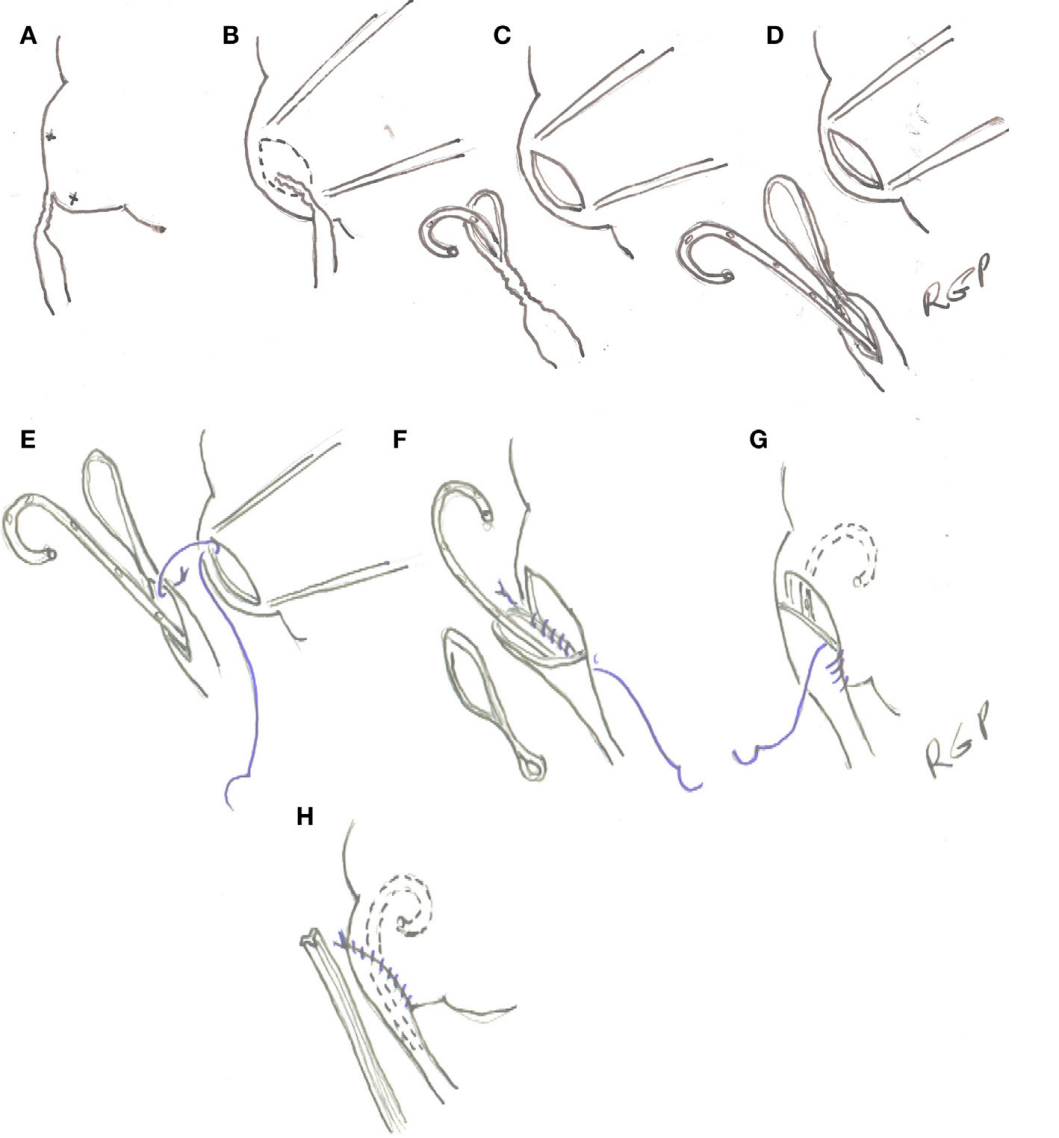
**(A)** Transparietal holding sutures. **(B,C)** Outline and incision in the renal pelvis. **(D)** Spatulation of ureter. **(E–H)** Ureteropelvic anastomosis with running suture starting in the posterior wall of the pelvis.

Once the area of the UPJ and proximal ureter is exposed and dissected free of surrounding tissues, percutaneous 4-0 polypropylene sutures are placed in the medial aspect of the renal above and below the UPJ to rotate anteriorly and stabilize it (Figures 3A,B). An ellipse of renal pelvis around the UPJ is incised and left attached to the upper ureter to serve as a handle (Figure 3C). The ureter is spatulated along its lateral aspect for an extension determined by the image of the retrograde pyelogram or until it is wide enough to allow the tip of an instrument to be introduced alongside the JJ stent (Figure 3D). Care is taken to avoid cutting the stent in these maneuvers. The anastomosis is performed with 5-0 Polyglecaprone 25 (Monocryl^®^) with 13 mm half circle needle (TF plus) (see text footnote 1) suture cut to 10 cm in length and introduced through the 5-mm port. A knot is tied at the end of the suture to facilitate its identification. A running suture is started in the cephalad part of the posterior wall of the open pelvis with the knot outside (Figure 3E). The suture is done from inside the renal pelvis and carried around the apex. At this point, the proximal end of the stent is placed in the renal pelvis and the suture of the anterior wall of pelvis and spatulated ureter run cephalad (Figures 3F–H). If the spatulated ureter does not exactly match the opening in the pelvis, a pelvis to pelvis suture is carried out. The needles can be removed through the 3-mm port under direct vision.

**Figure 4:**
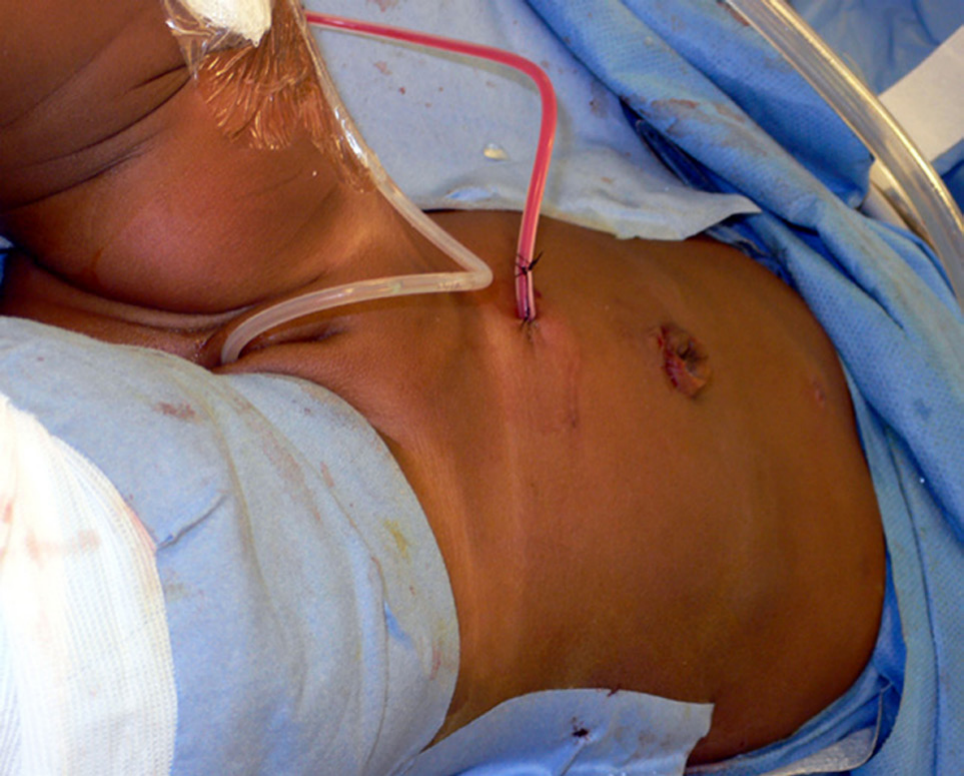
**Photograph showing bladder catheter and pararenal drain exiting from lower 3 mm port site**.

If at the end of the procedure doubts exist as to the distal position of the stent, X-ray of cystoscopic control is performed. A 10 Ch. Blake^®^ drain (see text footnote 1) is left in the vicinity of the anastomosis and brought out through the lower quadrant port (Figure 4).

### Postoperative Care

The child is fed after recovery from anesthesia. When the output from the Blake drain is minimal (usually after 24–48 h), the bladder catheter is removed. If no increase output from the drain is observed, it is removed and the child discharged from the hospital. The double J stent is removed under a brief general anesthetic 4–6 weeks after the operation. Renal ultrasounds are obtained at 1, 3, and 6 months after stent removal and at yearly intervals thereafter. Isotopic renography is done selectively if the dilatation persists on ultrasonography or there are other clinical indications.

## Results

Forty children underwent MLP between August 2010 and November 2015. Of these 24 (14 males) were 24 months or younger. No open pyeloplasties were performed during this time period. Two children had prior percutaneous nephrostomy and one a failed open pyeloplasty. Mean (range) follow-up after surgery was 18 months (3–55). Twenty-one children had prenatally detected hydronephrosis and three were discovered incidentally by ultrasonography. The mean (range) age at operation was 7.9 months (1–23) *and the median age 4 months*. The mean (range) weight was 7.4 kg (4–12). There was one conversion to open surgery for technical difficulties in a 2-month-old 4.3-kg girl with horseshoe kidney. Typical time for cystoscopy and stent placement was <30 min. Total operating time was mean (range) 214 min (93–360).

Preoperative SFU grade was 3.8 and mean (range) AP diameter 28 mm (10–50). After MLP the AP diameter was 9 mm (0–30) and SFU grade 1.4 (0–3). In nine patients, the dilatation resolved completely; and in 11, there was minimal residual dilatation (SFU grade 2) for an 83% resolution of hydronephrosis within the period of follow-up. In four patients, there was residual grade 3 SFU dilatation, all improved from a preoperative grade 4. There were no clinical manifestations, the AP diameter decreased in these four children from a mean of 40 mm (24–50) to 20 mm (13–30). Further analysis of children with persistent grade 3 dilatation revealed that the mean follow-up for this subgroup was 10 months (3–16). One of these children had also dilating reflux to the ureter distal to the obstruction due to reflux, which has since resolved. Another child had a hypoplastic ureter, which made stenting difficult. Despite the persistent dilatation, renal function has been maintained and the drainage curve on the diuresis renogram improved. No patient suffered stent-related complications. There was one omental prolapse through the lower quadrant port in a child in whom an Easy Flow drain^3^ was used and it had to be reduced under anesthesia. No drain-related complication was seen in the rest of the group. There were no postoperative urinary tract infections and no patient required re-intervention for obstruction. The placement of the 5-mm port in the umbilicus and the two additional minitrocars left virtually no visible abdominal scars.

## Discussion

Minilaparoscopy in infants presents unique technical challenges given the limited working space available and the occasional difficulty encountered in stenting the anastomosis. Although reported success of LP in children parallels or exceeds that of open surgery (9) the main advantage of MLP in infants is cosmetic since the 3-mm ports leave barely visible scars (10). Those who argue that in infants an open pyeloplasty can be done through a very small incision making MLP unnecessary (2, 3) seem to ignore the fact that scars grow with the body.

Our results are similar to other series reported in the literature with regard to success rate and operating time. We continue to use retrograde placement of the double J stent because we have had no stent-related postoperative complications with this method. The use of a perirenal drain offers the advantage of avoiding postoperative urine leak in the peritoneal cavity caused by a malfunctioning stent that often requires re-intervention. With the use of the Blake drain, we have had no drain-related complications. In one of our children, a *larger drain* was used in error and he developed an omental prolapse that had to be reduced under anesthesia. This complication has been reported by others using larger ports (1).

Although there are some reported series of LP in infants with minimal complications (11, 12); in other series, stent reported complications have ranged between 10 (1) and 35% of cases (13). Some authors reported acceptable results with unstented LP in selected cases (14) but others have published unfavorable results when no stents were used (15).

One report suggested that there were fewer complications with the use of a percutaneous transanastomotic stent (13), but in others, the results were similar or better compared with JJ stent (16). In our series with retrograde placement of the JJ stent after a retrograde pyelogram, there have been no stent-related postoperative complications.

The choice of trans- or retroperitoneal approach is surgeon dependent. In small children, the abdominal cavity provides a greater working space for suturing. Also, the need to make a 10- to 15-mm incision to develop the space in the retroperitoneal approach (17, 18) negates the cosmetic advantages of our approach.

Robotic pyeloplasty has also been applied to infants. In a recent series, including 60 patients from 6 centers, the success rate was 91%, the complication rate 11%, and the surgical time 232 min (19). Comparing these results along with the adverse cosmetic effect of the larger ports, the additional cost and limited availability of the equipment, it appears that MLP should be a preferred approach for young children with UPJO.

This study has the limitation of being retrospective and including a relatively small number of patients; however, our results and others previously published suggest to us that MLP *is an excellent procedure* for the treatment of UPJO in infants.

## Author Contributions

BL: performed operations, assisted with data collection, and preparation of manuscript. MB: performed some operations, assisted with data collection, and reviewed the manuscript. RG: data collection, writing manuscript and supervision of all operations.

## Conflict of Interest Statement

The authors declare that the research was conducted in the absence of any commercial or financial relationships that could be construed as a potential conflict of interest.

## Footnotes

^1^Ethicon US, LLC

^2^Karl Storz GmbH & Co. KG, Tuttlingen, Germany

^3^P.J.Dahlhausen & Co. GmbH, Cologne, Germany
